# A multicenter, randomized, double-blind trial comparing the efficacy and safety of TUDCA and UDCA in Chinese patients with primary biliary cholangitis

**DOI:** 10.1097/MD.0000000000005391

**Published:** 2016-11-28

**Authors:** Hong Ma, Minde Zeng, Ying Han, Huiping Yan, Hong Tang, Jifang Sheng, Heping Hu, Liufang Cheng, Qing Xie, Youfu Zhu, Guofeng Chen, Zhiliang Gao, Wen Xie, Jiyao Wang, Shanming Wu, Guiqiang Wang, Xiaohui Miao, Xiaoqing Fu, Liping Duan, Jie Xu, Lai Wei, Guangfeng Shi, Chengwei Chen, Minhu Chen, Qin Ning, Chen Yao, Jidong Jia

**Affiliations:** a Liver Research Center, Beijing Friendship Hospital, Capital Medical University, Beijing; b Gastroenterology Department, Renji Hospital, Shanghai Jiao Tong University, Shanghai; c Department of Gastroenterology, Xijing Hospital, Fourth Military Medical University, Xi’an; d Clinical Research Center for Autoimmune Liver Disease, Beijing You-an Hospital Capital Medical University, Beijing; e Department of Infectious Diseases, Huaxi Hospital, Chengdu; f Department of Infectious Diseases, Zhejiang University 1st Affiliated Hospital, Hangzhou; g Department of Comprehensive Treatment II, Eastern Hepatobiliary Surgery Hospital, Second Military Medical University, Shanghai; h Department of Gastroenterology and Hepatology, Chinese People's Liberation Army General Hospital, Beijing; i Department of Infectious Diseases, Ruijin Hospital, Shanghai; j Hepatology Department, Nanfang Hospital, Southern Medical University, Guangzhou; k Liver Fibrosis Noninvasive Diagnosis and Treatment Center, 302 Military Hospital, Beijing; l Department of Infectious Diseases, Sun Yat-Sen University 3rd Affiliated Hospital, Guangzhou; m Liver Disease Center, Beijing Ditan Hospital, Capital Medial University, Beijing; n Department of Gastroenterology and Hepatology, Zhongshan Hospital, Fudan University; o Shanghai Public Health Center, Shanghai; p Department of Infectious Disease, Peking University First Hospital, Beijing; q Department of Infectious Diseases, Changzheng Hospital, Second Military Medical University, Shanghai; r Department of Infectious Diseases, Hangzhou Sixth People's Hospital, Hangzhou; s Department of Gastroenterology, The 1st Affiliated Hospital of Kunming Medical University, Kunming, China; t Department of Infectious Diseases, The Third People's Hospital Affiliated to Shanghai Jiaotong University School of Medicine, Shanghai; u Hepatology Unit, Peking University People's Hospital, Beijing; v Department of Infectious Diseases, Shanghai Huashan Hospital; w Department of Infectious Diseases, 85th PLA Hospital, Shanghai; x Department of Gastroenterology, First Affiliated Hospital, Sun Yat-sen University, Guangzhou; y Institute and Department of Infectious Diseases, Tongji Hospital, Tongji Medical College, Huazhong University of Science and Technology, Wuhan; z Department of Biostatistics, Peking University First Hospital, Beijing, China.

**Keywords:** pimary biliary cholangitis, tauroursodeoxycholic acid, ursodeoxycholic acid

## Abstract

**Aim:**

Tauroursodeoxycholic acid (TUDCA) is a taurine conjugated form of ursodeoxycholic acid (UDCA) with higher hydrophility. To further evaluate the efficacy and safety of TUDCA for primary biliary cholangitis (PBC), we performed this study on Chinese patients.

**Methods:**

199 PBC patients were randomly assigned to either 250 mg TUDCA plus UDCA placebo or 250 mg UDCA plus TUDCA placebo, 3 times per day for 24 weeks. The primary endpoint was defined as percentage of patients achieving serum alkaline phosphatase (ALP) reduction of more than 25% from baseline.

**Results:**

At week 24, 75.97% of patients in the TUDCA group and 80.88% of patients in the UDCA group achieved a serum ALP reduction of more than 25% from baseline (*P* = 0.453). The percentage of patients with serum ALP levels declined more than 40% following 24 weeks of treatment was 55.81% in the TUDCA group and 52.94% in the UDCA group (*P* = 0.699). Both groups showed similar improvement in serum levels of ALP, aspartate aminotransferase, and total bilirubin (*P* > 0.05). The proportion of patients with pruritus/scratch increased from 1.43% to 10.00% in UDCA group, while there's no change in TUDCA group (*P* = 0.023). Both drugs were well tolerated, with comparable adverse event rates between the 2 groups.

**Conclusions:**

TUDCA is safe and as efficacious as UDCA for the treatment of PBC, and may be better to relieve symptoms than UDCA.

## Abstract 2

Supplemental Digital Content is available in the text

## 1 Introduction

Primary biliary cholangitis (PBC) is an immune-mediated chronic disease associated with progressive intrahepatic cholestasis. Although the pathogenesis of PBC remains incompletely understood, the accumulation of endogenous hydrophobic bile acids due to destruction of small bile ducts is thought to promote the progression of liver cell injury and finally lead to hepatic fibrosis and cirrhosis.^[1,2]^

Administration of ursodeoxycholic acid (UDCA) is the standard of care for PBC patients ^[3–5]^ and is recommended by major guidelines.^[6,7]^ The primary therapeutic action of hydrophilic UDCA is to replace hydrophobic bile acids, thereby attenuating cholestasis and hepatocellular injury. Tauroursodeoxycholic acid (TUDCA), a taurine conjugated form of UDCA with higher hydrophility, is the primary metabolite of UDCA.^[8–10]^ Setchell et al^[11]^ reported that the administration of TUDCA (500–1,500 mg daily) to patients with PBC led to an enrichment of UDCA by 34% to 41%, which resulted in a greater shift toward a more hydrophilic bile acid pool. A dose–response study indicated that TUDCA was at least as effective as UDCA for PBC, and this result was confirmed by a crossover study.^[8]^

However, no large-scale, randomized, clinical trial has compared the efficacy and safety of TUDCA and UDCA for PBC patients. Therefore, we performed this multicenter, randomized, double-blind, controlled study to evaluate the efficacy and safety of these 2 agents in Chinese patients with PBC.

## 2 Methods

### 2.1 Patient selection

Eligible patients were Chinese males or females with PBC, aged 18 to 70 years. The inclusion criteria included the following: serum alkaline phosphatase (ALP) levels of 2 times or more the upper limit of normal (ULN, defined as 150 IU/L); serum positive for antimitochondrial antibodies (AMA, detected by indirect immunofluorescent assay, Euroimmun, Inc.) and/or anti-AMA-M2 (anti-PDC-E2) antibodies; and liver histologic features of PBC.

The exclusion criteria included the following: previous treatment with UDCA, corticosteroids or other immunomodulators; evidence of extrahepatic biliary obstruction; hepatitis B and/or hepatitis C virus infection; history or current evidence of decompensated liver disease; body mass index (BMI) more than 28 (kg/m^2^); pregnancy or breastfeeding; unwillingness to use a double-barrier method of contraception; addiction to alcohol or illicit drugs within the past year; drug-induced liver diseases; hepatocellular carcinoma or other malignancy requiring treatment; other serious medical conditions that might confound efficacy or safety assessments; and organ transplant or planned organ transplantation.

The laboratory exclusion criteria included the following: hemoglobin less than 11 g/dL for men and less than 10 g/dL for women, white blood cell count less than 3000/mm^3^, neutrophil count less than 1500/mm^3^, platelet count less than 50,000/mm^3^, and serum albumin less than 3.3 g/dL; alanine aminotransferase (ALT) 10 times or more ULN and/or aspartate aminotransferase (AST) 10 times or more ULN; ALT 5 times or more ULN and/or AST 5 times or more ULN with immunoglobulin G (IgG) 2 times or more ULN; total bilirubin (T-Bil) 4 times or more ULN; prothrombin time prolonged by 3 seconds over the upper limit of the reference value or prothrombin time activity (PTA) 60% or less; serum creatinine (SCr) 1.5 times or less ULN; and serum alpha-fetoprotein more than 100 ng/mL.

### 2.2 Study design

This multicenter, double-blind, randomized phase III trial was designed to assess the efficacy and safety of TUDCA versus UDCA treatment for 24 weeks in Chinese adults with PBC. A centralized telecommunication-based interactive voice response system was used for patient randomization after patient eligibility was determined through clinical and laboratory screening assessments. Eligible patients were randomly assigned to the TUDCA or UDCA group at a ratio of 2:1.

Patients in the TUDCA group were treated with TUDCA plus UDCA placebo at a dose of 250 mg 3 times daily; patients in the UDCA group were treated UDCA plus TUDCA placebo at a dose of 250 mg 3 times daily. The first dose was administered at the baseline visit, and the patients were followed up at weeks 4, 12, and 24. At each visit, blood biochemistry, routine hematological tests, adverse events, and concurrent medications were assessed.

This trial was conducted with approval from the Chinese State Food and Drug Administration. The trial protocol was approved by the ethics committee at each participating center, and all patients provided written informed consent. The clinical data were collected and monitored in accordance with standardized data management and quality assurance procedures.

### 2.3 Efficacy and safety endpoints

The primary efficacy endpoint was the proportion of patients with serum ALP reductions greater than 25% from baseline following 24 weeks of treatment, and the proportion of patients with serum ALP reduction ns greater than 40% were also calculated referring to Barcelona Criteria. Secondary efficacy measurements included reductions in ALT, AST, ALP, GGT, and T-Bil from baseline levels following 24 weeks of treatment. Safety endpoints were assessed in the combined intent-to-treat population and included serious and non-serious adverse events and graded laboratory abnormalities.

### 2.4 Statistical analysis

This test uses positive controlled, noninferiority hypothesis testing. Test level was 0.025 for the unilateral test, with power set of 80%. Noninferiority margin, taking 10%, according to the samples estimated nQuery Advisor^®^, version 6.01 (Statistical Solutions, Boston MA, USA), the final estimate of sample size required for the test groups: control group = 112:56.

Statistical analyses were performed with SAS software (verion 9.4, North Carolina State University, NL). All tests were 2-sided; *P* values less than 0.05 were considered significant. The measurement data between groups were analyzed with a Student *t* test and Wilcoxon test. The categorical data between groups were analyzed with the chi-square test or Fisher exact probability test. The ranked data between groups were analyzed with the Wilcoxon test.

The clinical efficacy was evaluated at week 24. The Cochran-Mantel Haensze chi-square test was also applied. The 95% confidence intervals were utilized to determine whether the non-inferiority was greater than 10%.

## 3 Results

### 3.1 Patient enrollment

**Figure 1:**
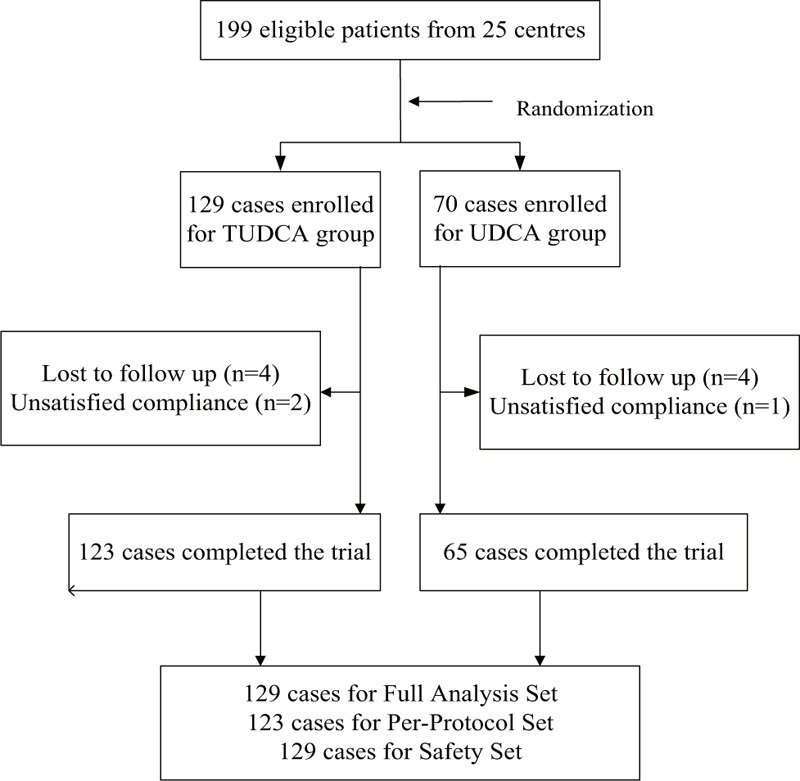
Flow chart of the participants.

A total of 199 Chinese patients with PBC comprised the intent-to-treat population. In total, 129 patients were randomized to receive TUDCA, and 70 patients to receive UDCA. During the study, 8 patients (4 in each group) dropped out of the trial: 1 patient (in the UDCA group) was lost after 4 weeks, 4 patients (2 in each group) after 12 weeks, 3 patients (1 in the TUDCA group and 2 in the UDCA group) after 24 weeks (Fig. 1).

### 3.2 Demographic and baseline characteristics of the patients with PBC

The demographic and baseline characteristics, including age, gender, BMI, and severity of cholestasis, were not significantly different between the TUDCA and UDCA groups. The patients in both groups had comparable Mayo risk scores (mean ± SD): 4.40 ± 0.80 in TUDCA group and 4.63 ± 0.81 in the UDCA group (*P* = 0.059). The aminotransferase-to-platelet ratio index of patients in 2 groups was also calculated: 1.99 ± 1.39 of TUDCA group, 1.79 ± 1.10 of UDCA group (*P* = 0.167), without significant difference, Forns score of UDCA group (10.20 ± 1.77) and TUDCA group (10.05 ± 1.69) were also comparable (*P* = 0.456). The AMA and AMA-M2 positive rates in each group were also similar. The patients in TUDCA took a mean dose of 13.69 ± 2.38 mg/kg/d, and the UDCA group was 13.15 ± 2.92 mg/kg/d (Table 1); this was in line with recommendations by the AASLD and EASL guidelines (12–15 mg per kg per day).^[6,7]^

**Table 1:**
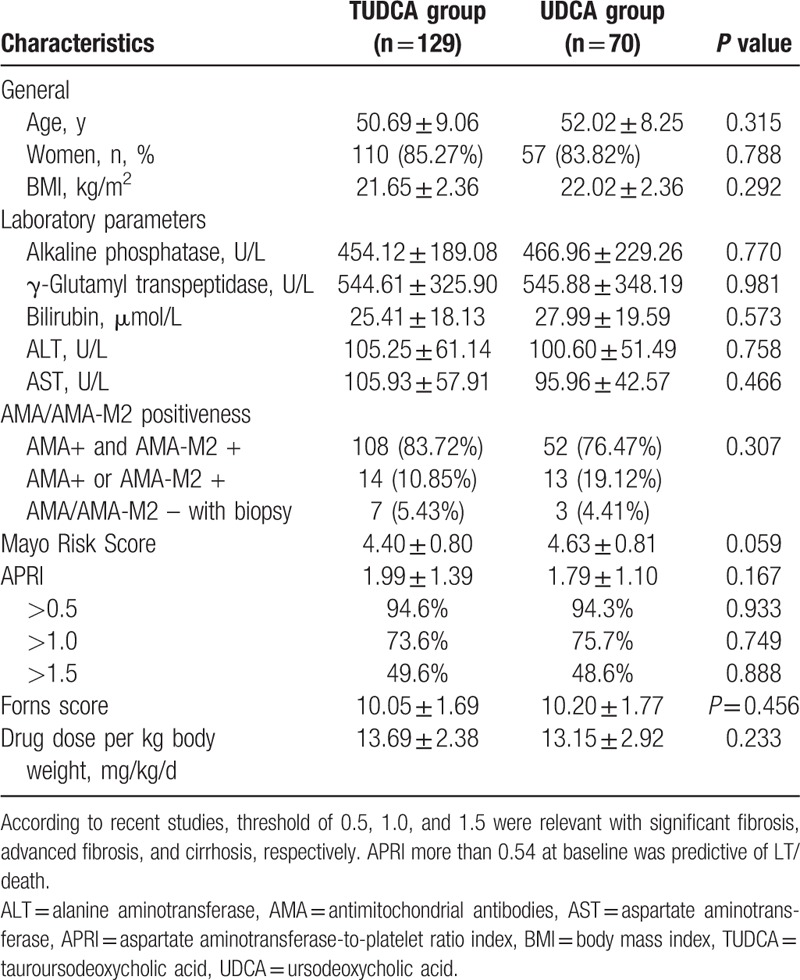
Baseline characteristics and disease features of the TUDCA and UDCA groups.

### 3.3 Biochemical responses

**Figure 2:**
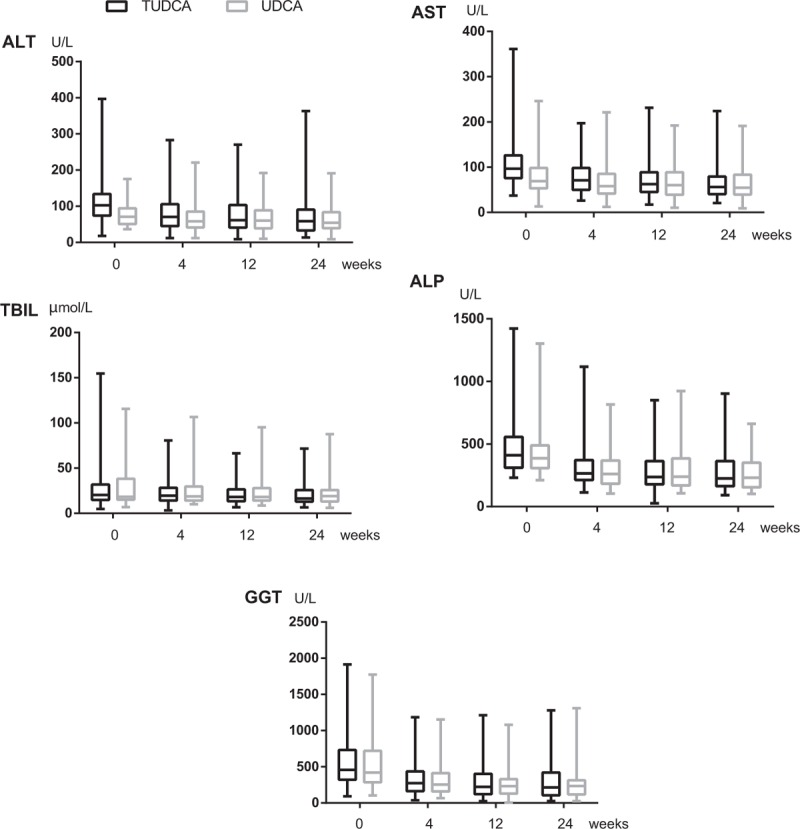
Changes in serum markers, including ALP, GGT, T-BIL, ALT, and AST, during the administration of TUDCA and UDCA groups. ALP = alkaline phosphatase, ALT = alanine aminotransferase, AST = aspartate aminotransferase, GGT = gamma glutamyl transpeptidase, T-Bil = total bilirubin, TUDCA = tauroursodeoxycholic acid, UDCA = ursodeoxycholic acid.

The time course of the changes in serum biochemical variables is presented in Fig. 2. As the primary efficacy endpoint, 76% of patients in the TUDCA group and 81% of patients in the UDCA group achieved a serum ALP reduction greater than 25% from baseline level following 24 weeks of treatment (*P* = 0.453). Similarly, at week 24, the proportion of patients who achieved a greater than 40% reduction in ALP levels from baseline was 56% and 53% in the TUDCA and UDCA groups, respectively (*P* = 0.699).

At week 24, the reduction in ALP, AST, and T-Bil levels from baseline in TUDCA-treated patients was also similar to that in UDCA-treated patients (*P* = 0.126, *P* = 0.274, and *P* = 0.107, respectively). However, the reductions in ALT and GGT from baseline were greater in UDCA than TUDCA treated patients (*P* = 0.029 and *P* = 0.027, respectively).

### 3.4 Symptoms and signs during treatment

**Figure 3:**
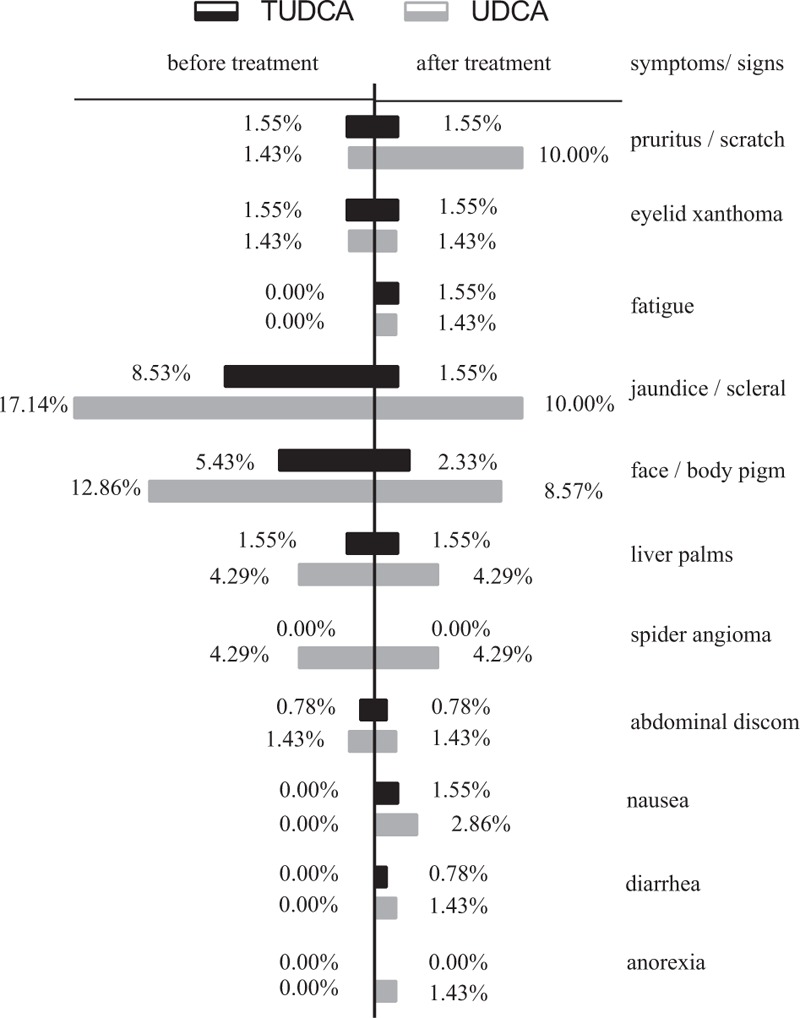
Symptoms and signs before and post treatment of TUDCA and UDCA groups: the proportion of patients with symptoms/signs of each group. TUDCA = tauroursodeoxycholic acid, UDCA = ursodeoxycholic acid.

Symptoms and signs reported by patients during the treatment include: pruritus/scratch, fatigue, abdominal discomfort, nausea, diarrhea and anorexia, eyelid xanthoma/xanthoma, jaundice/scleral yellow dye, face/body pigment, liver palms, and spider angioma. At baseline there were 26 cases (19 patients) of symptoms and signs in TUDCA group, and 30 cases (19 patients) of those in UDCA group. It turned out to be 18 cases (14 patients) of symptoms and signs in TUDCA group after treatment, and 33 cases (24 patients) in UDCA group of that (Fig. 3, Supplementary Table 3). Among all the symptoms/signs, 48.57% in TUDCA group have improved after treatment, while only 25.58% in UDCA group have improved (*P* = 0.035). It is noteworthy that the proportion of patients with pruritus/scratch increased from 1.43% to 10.00% in UDCA group, while there's no change in TUDCA group (*P* = 0.023). In terms of jaundice/scleral yellow dye, the proportion of patients decreased from 8.53% to 1.55% in TUDCA group, and from 17.14% to 10.00% in UDCA group (*P* = 0.049).

### 3.5 Safety and tolerability

Both drugs were generally well tolerated. Adverse events were found in 44% and 40% of the patients in TUDCA and UDCA groups, respectively (*P* = 0.55), most of them (93.80%) being considered not related to study medications (Table 2, Supplementary Table 4). In the TUDCA group only 5 adverse events (4 patients, 3.1%) were considered to be study drug related: diarrhea in 1 case, pruritus in 2 cases, rash in 1 case and dysmenorrheal in 1 case. In the UDCA group, only 3 adverse events (3 patients, 4.4%) were considered to be study drug related: rash in 1 case and nausea in 2 cases. No serious adverse events occurred during the study period.

**Table 2:**
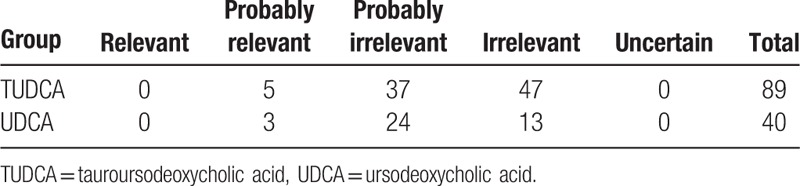
The relevance with medication of adverse effects in TUDCA and UDCA group.

## 4 Discussion

In this multicenter, double-blind, randomized trial study, a 24-week treatment with TUDCA or UDCA showed similar biochemical improvement (decline in ALP levels from baseline in 76% and 81% patients for TUDCA and UDCA groups, respectively) in PBC patients, demonstrating that TUDCA was not inferior to UDCA for this disease.

We defined the efficacy endpoint as percentage of patients with a 25% or more decline in ALP levels at 24 weeks of treatment, considering the commonly used Barcelona criteria of optimal response at 12 months treatment requires a decline in ALP more than 40% from baseline.^[12]^ For comparison, we also calculated the decline in ALP more than 40% from baseline at 24 weeks and found that more than half of the patients achieved this biochemical response in both groups. One of the limitations of our study was the relatively short duration of treatment (only 24 weeks). However, a recent report by Zhang et al^[13]^ demonstrated that biochemical response at the first sixth months of UDCA treatment had a similar predictability to that at 12 months of UDCA therapy, regarding the risk of poor outcomes such as complications of cirrhosis, liver-related death or liver transplantation. Therefore, we believe the results of our study could reflect the efficacy of longer term.

TUDCA is available on the market in these countries: Italy, Turkey, and China. As a more hydrophilic bile acid, TUDCA is a promising agent for patients with PBC,^[9]^ since endogenous hydrophobic bile acids play a major in progression of cholestatic liver injury.^[14–16]^ Previous studies showed that during TUDCA treatment significantly decreases cheno-deoxycholate and cholate in the serum, and lithocholic acid in the duodenal bile.^[11]^ This more profound shift toward a more hydrophilic bile acid pool by administrating TUDCA may be more favorable, leading to more efficient extraction of taurine-conjugated bile acids.^[8,17]^ Indeed, one study published recently shown that TUDCA (750 mg/d) is safe and more effective than UDCA (750 mg/d) in improving biochemical parameters in a 6-month double-blind randomized control trial for cirrhosis patients.^[18]^

It is also noteworthy that the same amount (750 mg) of TUDCA and UDCA per day is not bioequivalent because of their different molecular weight, and 500 mg TUDCA is approximately 375 mg UDCA in terms of bioequivalence. So it could be anticipated that TUDCA could be more effective than UDCA if given the same bioequivalent amount of drugs for PBC patients. It is also worth noting that 750 mg TUDCA and 750 mg UDCA are not equivalent molar amounts, since 500 mg TUDCA is approximately 375 mg UDCA in terms of bioequivalence.^[8]^ Thus, patients in the TUDCA group actually received smaller molar amounts of the drug and this may partially explain why TUDCA was not superior to UDCA in this study, since it is known that low-dose (<10 mg/kg/d) UDCA does not work well in patients with PBC.^[19]^

In conclusion, our current randomized, double-blind, controlled trial confirmed that TUDCA is as effective and safe as UDCA for patients with PBC.

## Acknowledgments

We appreciate the support and assistance provided by all the people who contributed to the study and this article including: Xiaojuan Ou, Yiwen Shi (Beijing Friendship hospital), Yanmin Liu (Beijing You-an Hospital), Hong Zhao (Beijing Ditan Hospital), Ping Han (302 Military Hospital), Rongbin Guo, Yunsheng Yang (301 Military Hospital), Jun Li (Peking University First Hospital), Bo Feng (Peking University People's Hospital), Gui Jia (Xijing Hospital, Fourth Military Medical University), Yimin Mao, Xiong Ma, Dekai Qiu (Shanghai Renji Hospital), Chuantao Tu (Zhongshan Hospital, Fudan University), Jianming Zheng (Shanghai Huashan Hospital), Qing Guo (Shanghai Ruijin Hospital), Ling Xu (Changzheng Hospital, Second Military Medical University), Xiaojin Wang (85th PLA Hospital), Dongxun Zhou (Eastern Hepatobiliary Surgery Hospital), Guoguang Xu (Shanghai Public Health Center), Bin Zhao (The Third People's Hospital Affiliated to Shanghai Jiaotong University School of Medicine), Jinhui Wang (First Affiliated Hospital, Sun Yat-sen University), Bingliang Lin (Sun Yat-Sen University 3rd Affiliated Hospital), Xiaolin Zhu, Yingying Zhang (Nanfang Hospital, Southern Medical University), Ke Ma (Wuhan Tongji Hospital), Lichun Wang (Sichuan Huaxi Hospital), Xiangqian Dong (The 1st Affiliated Hospital of Kunming Medical University), Guoping Sheng (Zhejiang University 1st Affiliated Hospital), Chuntao Liu (Hangzhou Sixth People's Hospital), and Yanxiao Yan (Department of Biostatistics, Peking University First Hospital) who provided statistical consultation.

## Supplementary Material

Supplemental Digital Content
