# Supplementary material for: A multicenter, randomized, double-blind trial comparing the efficacy and safety of TUDCA and UDCA in Chinese patients with primary biliary cholangitis
Source: Medicine (Baltimore). 2016 Nov 28;95(47):e5391. doi: 10.1097/MD.0000000000005391 (PMC5134868; doi:10.1097/MD.0000000000005391)
Supplement: Supplemental Digital Content [file medi-95-e5391-s001.docx]

Table 3. Symptoms and signs before and post treatment:

| **Group** | **Patient ID** | **Symptoms before treatment** | **Symptoms after treatment** | **Change** |
| --- | --- | --- | --- | --- |
| TUDCA | 302 | face pigmentation | - | improved |
|  | 302 | liver palms | liver palms | no change |
|  | 302 | - | nausea | worse |
|  | 109 | - | nausea | worse |
|  | 109 | - | liver discomfort | worse |
|  | 114 | abdominal pain | - | improved |
|  | 93 | pigment | - | improved |
|  | 93 | - | scratch | worse |
|  | 62 | scleral yellow dye | - | improved |
|  | 46 | - | stomach discomfort | worse |
|  | 204 | xanthoma | xanthoma | no change |
|  | 204 | face pigmentation | face pigmentation | no change |
|  | 226 | light liver palm | light liver palm | no change |
|  | 226 | scleral yellow dye | - | improved |
|  | 226 | jaundice | - | improved |
|  | 108 | - | fatigue | worse |
|  | 165 | face pigmentation | face pigmentation | no change |
|  | 149 | - | pruritus | worse |
|  | 56 | face pigmentation | - | improved |
|  | 77 | - | fatigue | worse |
|  | 225 | face pigmentation | - | improved |
|  | 53 | scleral yellow dye | - | improved |
|  | 100 | eyelid lipoma | eyelid lipoma | no change |
|  | 100 | jaundice | - | improved |
|  | 100 | scleral yellow dye | - | improved |
|  | 318 | pruritus | - | improved |
|  | 43 | jaundice | - | improved |
|  | 263 | face pigmentation | - | improved |
|  | 134 | jaundice | jaundice | no change |
|  | 11 | jaundice | - | improved |
|  | 11 | - | diarrhea | worse |
|  | 76 | pruritus | - | improved |
|  | 213 | jaundice | - | improved |
|  | 16 | jaundice | jaundice | no change |
|  | 16 | pigment | pigment | no change |
| UDCA | 124 | jaundice | jaundice | no change |
|  | 124 | pigment | pigment | no change |
|  | 124 | liver palm | liver palm | no change |
|  | 124 | spider angioma | spider angioma | no change |
|  | 177 | face pigmentation | light face pigmentation | improved |
|  | 136 | - | nausea | worse |
|  | 86 | face pigmentation | face pigmentation | no change |
|  | 167 | scleral yellow dye | - | improved |
|  | 95 | - | nausea | worse |
|  | 307 | - | pruritus | worse |
|  | 158 | scleral yellow dye | scleral yellow dye | no change |
|  | 158 | pigmentation | pigmentation | no change |
|  | 32 | - | anorexia | worse |
|  | 32 | - | pruritus | worse |
|  | 162 | scleral yellow dye | - | improved |
|  | 208 | - | pruritus | worse |
|  | 94 | - | abdominal distension | worse |
|  | 184 | light jaundice | jaundice | worse |
|  | 188 | - | fatigue | worse |
|  | 256 | - | pruritus | worse |
|  | 141 | jaundice | jaundice | no change |
|  | 141 | - | pruritus | worse |
|  | 320 | - | pruritus | worse |
|  | 129 | pigmentation | pigmentation | no change |
|  | 153 | jaundice | jaundice | no change |
|  | 115 | jaundice | - | improved |
|  | 115 | pigmentation | - | improved |
|  | 54 | - | diarrhea | worse |
|  | 65 | scleral yellow dye | - | improved |
|  | 65 | liver palm | liver palm | no change |
|  | 65 | spider angioma | spider angioma | no change |
|  | 89 | - | pruritus | worse |
|  | 262 | scleral yellow dye | - | improved |
|  | 68 | pigmentation | - | improved |
|  | 68 | pruritus | - | improved |
|  | 203 | scleral yellow dye | scleral yellow dye | no change |
|  | 203 | pigmentation | pigmentation | no change |
|  | 269 | face pigmentation | - | improved |
|  | 175 | eyelid xanthoma | eyelid xanthoma | no change |
|  | 119 | light jaundice | light jaundice | no change |
|  | 12 | abdominal pain | - | improved |
|  | 12 | spider angioma | spider angioma | no change |
|  | 12 | liver palm | liver palm | no change |

Table 4 The observed adverse events by System Organ Class after 6-month TUDCA and UDCA Treatment.

| **System Organ Class** | **n** | **UDCA** | **TUDCA** |
| --- | --- | --- | --- |
| Investigations | 31 | 10 | 21 |
| Respiratory, thoracic and mediastinal disorders | 16 | 2 | 14 |
| Gastrointestinal disorders | 15 | 9 | 6 |
| Skin and subcutaneous tissue disorders | 15 | 5 | 10 |
| Hepatobiliary disorders | 13 | 3 | 10 |
| Cardiac disorders | 8 | 1 | 7 |
| Renal and urinary disorders | 8 | 4 | 4 |
| Blood and lymphatic system disorders | 6 | 1 | 5 |
| General disorders and administration site conditions | 4 | 3 | 1 |
| Nervous system disorders | 4 | 0 | 4 |
| Musculoskeletal and connective tissue disorders | 3 | 2 | 1 |
| Eye disorders | 2 | 0 | 2 |
| Injury, poisoning and procedural complications | 1 | 0 | 1 |
| Metabolism and nutrition disorders | 1 | 0 | 1 |
| Reproductive system and breast disorders | 1 | 0 | 1 |
| Total | 128 | 40 | 88 |
